# Aberrant personal space is associated with paranoia, altered stress regulation, and unfavourable outcomes at 6 months’ follow-up in schizophrenia

**DOI:** 10.1038/s41380-025-02999-x

**Published:** 2025-04-09

**Authors:** Adamantini Hatzipanayioti, Sebastian Walther, Nicole Gangl, Frauke Conring, Florian Wüthrich, Katharina Stegmayer

**Affiliations:** 1 Translational Research Center, University Hospital of Psychiatry and Psychotherapy, University of Bern, Bern, Switzerland; 2 Department of Psychiatry, Psychosomatics, and Psychotherapy, Center for Mental Health, University of Würzburg, Würzburg, Germany

**Keywords:** Predictive markers, Physiology

## Abstract

Paranoia is a central feature of schizophrenia and linked with poor outcomes. Paranoid experience is sometimes hard to identify in the clinical interview. In contrast, personal space (PS) measures detected patients with paranoia with excellent sensitivity and specificity. Here we test whether we can substantiate aberrant PS regulation in paranoia and whether PS was associated with stress markers and longitudinal outcomes. We included 144 participants (92 patients with schizophrenia spectrum disorders and 52 age and sex-matched healthy controls). We measured PS and stress markers during two behavioural tasks on interpersonal distance. In addition, we assessed social outcomes at baseline and after 6 months. Data corroborated that paranoia increased PS. Moreover, we confirmed that PS detected paranoia with excellent sensitivity (92%) at 1.1 m, and severe paranoia with 87% sensitivity and 81% specificity at 1.6 m. In addition, stress (Electrodermal activity) during the PS task was associated with paranoia and PS. Furthermore, higher stress at baseline predicted less improvement of social outcome after 6 months. Finally, improvement of PS over 6 months was associated with improvement of social functioning. PS may indeed serve as a simple bedside test for paranoia. Furthermore, results have direct implications in clinical practice as they suggest that it is advisable to maintain increased PS with paranoid patients. In addition, altered stress regulation and persistently increased PS may indicate unfavourable outcomes in the short-term follow-up. Thus, patients with persistently increased PS may benefit from special therapeutic attention.

## Introduction

Paranoia, “the unfounded fear that others intend to harm the individual”, is a central and disabling experience of schizophrenia [1]. Paranoia is associated with poverty, poor physical health, aggressive behaviour, suicidal ideation, and poor social outcome [2, 3]. Hitherto, paranoid threat is sometimes hard to detect in the clinical interview. In fact, patients with paranoia might cover persecutory delusions in clinical interviews as they tend to consider fewer individuals as trustworthy [4]. As a solution to this problem we proposed a simple bedside test to identify paranoid threat with excellent sensitivity (93%) and specificity (83%) at 1.1 m: the personal space test [5].

Personal space (PS) is the safe area around us causing discomfort and in some instances flight reactions when violated by others [6]. PS, also termed body-buffer zone or safety seeking area can be distinguished from peripersonal space, which refers to a reachable space that surrounds the body where the physical interaction between the self and objects in the environment occurs, for review see [7]. However, personal space was linked to the representation of peripersonal space [8]. PS is influenced by several factors, including gender, age, social status, and cultural norms [9–11]. Nevertheless, when many of these factors are controlled, PS remains remarkably stable across repeated measurements [12, 13]. Growing evidence suggests a pivotal relationship of increased PS and paranoia. In fact, in healthy individuals threat induces increased PS and invasion into the PS leads to paranoid ideation [14, 15]. Furthermore, schizophrenia patients have increased PS [5, 14, 16] (for review see [17]). Notably, we previously reported paranoid threat to be associated with abnormal PS regulation. Patients without paranoia were not different from controls in PS but experienced less comfort in the same study [5]. Likewise, one study showed a preference for greater PS associated with hallucinations [18]. In contrast, the literature is less clear on negative symptoms: greater distance indicated more severe [19], or less severe negative symptoms [16, 20]. Furthermore steeper peripersonal space boundaries correlated with the severity of persecutory delusions [21].

Critically, intrusion of the preferred PS seems to trigger stress. When perceived safety is compromised, we expect stress to increase in paranoia. In particular, the effect of social stress on paranoid ideation has been emphasized. Thus, a cyclical relationship between increased need of PS, paranoia, and stress may apply where paranoia increases PS, intrusion into PS causes stress, and increased stress elicits more paranoia. However, studies with physiological stress markers are scarce, data remains equivocal [22], and mechanisms translating stress into paranoia are not fully established yet. Whether stress reactions contribute to PS regulation in patients with paranoia remains currently unknown.

Finally, the field struggles to understand how paranoia precisely relates to poor social outcome in schizophrenia. Previous reports show that paranoia is associated with social functioning [4, 23], social withdrawal, isolation [24], and feelings of loneliness [25, 26]. Therefore, we may speculate that PS critically contributes to social withdrawal and poor functional outcome in patients with paranoia.

The current study tested whether we could corroborate that impaired PS regulation may detect paranoia among patients with schizophrenia. Based on our initial finding we hypothesized that increased PS > 1.1 m can detect paranoia with high sensitivity. Furthermore, we tested whether impaired PS regulation and paranoia are associated with markers of stress. Here, we hypothesized that paranoia as well as increased PS are associated with altered stress regulation. Finally, we tested whether impaired PS as well as markers of stress are predictive of functional outcome after 6 months. We hypothesized impaired PS as well as altered stress regulation at baseline to be associated with poor functioning at 6 months follow-up.

## Methods

### Participants

92 patients with schizophrenia and 52 age and gender matched healthy controls participated.

Data from our previous study on personal space was used for statistical power calculations using G*power software. We recruited patients from the University Hospital of Psychiatry and Psychotherapy in Bern, Switzerland, and controls from the community. Exclusion criteria for all participants were substance abuse or dependence other than nicotine and additional for controls history of any psychiatric disorder as well as first degree relatives with schizophrenia spectrum disorders. All participants provided written informed consent. Protocols and procedures adhered to the declaration of Helsinki and were approved by the local Ethics Committee (KEK 2016-00166).

Clinical diagnosis was evaluated with the Diagnostic and Statistical Manual of Mental Disorders (DSM-5). To screen psychiatric disorders in controls we completed the Mini International Neuropsychiatric Interview [27]. All but eight patients received antipsychotic medication. We calculated olanzapine (OLZ) equivalents according to Leucht and colleagues [28]. Five participants (3 patients, 2 controls) were removed from further analysis due to extreme values in stop-distance and stress measures. For demographics and clinical characteristics see Table 1.

**Table 1:** Demographic and clinical characteristics, mean (SD).

Values	Controls (*N* = 50)	Patients (*N* = 89)	*Χ* ^*2*^*/ t*	*P* value
Male, n (%)	32 (64)	55 (61.8)	0.066	0.86
Age, mean (SD), years	35.38 (12.26)	38.29 (12.82)	−1.32	0.19
Education, mean (SD), years	17.44 (3.64)	13.48 (2.86)	6.55	**<0.001**
BMI, mean (SD), kg/m^2^	24.55 (3.44)	25.15 (4.56)	−0.87	0.38
GPTS, mean (SD)	17.51 (3.14)	36.59 (19.20)	−8.42	**<0.001**
Distance, mean (SD), m	1.17 (0.37)	2.01 (1.17)	29.38	<**0.001**
GAF, mean (SD)	85.98 (5.53)	41.29 (15.24)	18.98	<**0.001**
SOFAS, mean (SD)	85.93 (5.37)	41.05 (15.56)	24.14	<**0.001**
OLZ, mean (SD), mg		13.7 (10.47)		
Illness Duration, mean (SD), years		9.42 (9.25)		

Green paranoid thought scale					
Values	Controls (*N* = 50)	Paranoid (*N* = 35)	Non -paranoid (*N* = 40)	*Χ* ^*2*^*/ F*	*P* value
Male, n (%)	32 (64)	20 (57.1)	25 (62.5)	0.42	0.80
Age, mean (SD), years	35.38 (12.26)	40.34 (11.24)	34.63 (12.74)	2.44	0.09
Education, mean (SD), years	17.44 (3.64)	13.72 (2.99)	13.57 (2.74)	21.02	**<0.001**
BMI, mean (SD), kg/m^2^	24.55 (3.44)	25.51 (5.17)	25.06 (4.51)	0.52	0.59
GPTS, mean (SD)	17.51 (3.14)	54.37 (12.71)	21.03 (5.19)	265.77	**<0.001**
Distance, mean (SD), m	1.17 (0.37)	2.32 (1.42)	1.73 (0.83)	15.04	<**0.001**
GAF, mean (SD)	85.98 (5.53)	34.59 (12.63)	48.18 (14.29)	229.43	<**0.001**
SOFAS, mean (SD)	85.93 (5.37)	36.38 (11.79)	47.83 (16.12)	199.38	<**0.001**
OLZ, mean (SD), mg		18.09 (12.00)	9.52 (7.96)	6.19	<**0.001**
Illness Duration, mean (SD), years		12.21 (10.20)	5.73 (6.19)	10.40	<**0.001**

Bern psychopathology scale						
Values	Controls (*N* = 50)	Paranoid Threat (*N* = 29)	Non-paranoid (*N* = 44)	Paranoid power (*N* = 15)	*Χ* ^*2*^*/ F*	*P* value
Male, n (%)	32 (64)	17 (58.6)	26 (59.1)	12 (80)	2.40	0.49
Age, mean (SD), years	35.38 (12.26)	41.31 (12.59)	38.14 (12.92)	32.33 (11.83)	2.23	0.08
Education, mean (SD), years	17.44 (3.64)	13.84 (3.49)	13.23 (2.35)	13.78 (2.91)	16.10	**<0.001**
BMI, mean (SD), kg/m^2^	24.55 (3.44)	24.34 (4.75)	25.70 (4.38)	25.14 (4.92)	0.82	0.48
GPTS, mean (SD)	17.51 (3.14)	45.26 (21.33)	30.74 (16.87)	36.50 (15.21)	4.63	**0.01**
Distance, mean (SD), m	1.17 (0.37)	2.56 (1.18)	1.68 (0.90)	1.85 (1.57)	12.90	**<0.001**
GAF, mean (SD)	85.98 (5.53)	39.22 (13.83)	45.51 (15.09)	35.50 (15.34)	2.67	0.07

Values (continued)	Controls (N = 50)	Paranoid Threat (N = 29)	Non-paranoid (N = 44)	Paranoid Power (N = 15)	*Χ* ^*2*^*/ F*	*P* value
SOFAS, mean (SD)	85.93 (5.37)	38.74 (12.34)	45.85 (15.40)	39.25 (18.79)	1.94	0.15
OLZ, mean (SD), mg		14.15 (9.33)	12.79 (12.67)	14.46 (7.78)	0.16	0.84
Illness Duration, mean (SD), years		11.73 (8.93)	8.32 (9.08)	3.12 (3.76)	2.27	**0.02**

Significant *p* values are indicated in bold.

*BMI* body mass index, *GPTS* green paranoid thoughts scale, *GAF* global assessment of functioning scale, *SOFAS* social assessment of functioning scale, *OLZ* olanzapine equivalents.

### Assessments at baseline and 6month follow-up

Symptom severity was assessed with the Positive and Negative Syndrome Scale (PANSS [29]), the Green Paranoid Thought Scale (GPTS [30]), and the Bern Psychopathology Scale (BPS [31]). The GPTS is a self-report measuring the severity of paranoia. Scores in Persecution (Part B) range from 16 (no paranoia) to 80 (severe paranoia). Based on the recommended cut-off score, participants were categorized with severe paranoia (score above 35) and without or mild paranoia [32]. In addition, patients were classified into patients with paranoid threat, paranoid power (i.e. patients with paranoid experience of power and grandiosity), and no paranoia according to the BPS. The BPS rates paranoia on a seven-point Likert scale ranging from severe paranoid threat (−3) to severe paranoid power (+3), while 0 represents no paranoia. The expert ratings consider objective signs (i.e. sweating or pupil width), indirect signs (i.e. emotional content of delusions) as well as subjective experience of paranoid threat or power. In addition, we assessed functional outcome with the Social and Occupational Functioning scale (SOFAS [33]) and the Global Assessment of Functioning (GAF [34]). Clinical ratings were performed blind for results of the PS task.

To assess PS regulation we used two behavioral tasks: the stop-distance task measuring the preferred PS between a male, unfamiliar experimenter (FW) and the participant, and the fixed-distance paradigm assessing participants’ subjective comfort at given PS of 0.5, 1, 1.5, 2 and 2.5 meters with a visual analog scale (VAS; range: 0–100; 0 extreme discomfort) [5].

During the stop-distance task, participants indicate the minimum tolerable distance while distance is varied in four conditions each repeated three times in a random order. In detail, during the Active Approach condition, participants were instructed to walk towards the experimenter starting from a distance of 7 m, facing the experimenter, and to stop when they start to feel uncomfortable. In the Passive Approach condition, the experimenter walked towards the participants who indicated when the experimenter reached a distance, they start to feel uncomfortable. Both conditions were carried out with and without eye-contact.

To measure stress during the experimental paradigms we assessed psychophysiological indicators of stress (frequency of Skin Conductance Responses of electrodermal activity per minute, SCR [35]) with the E4 wireless wristband (Empatica Inc., MIT Media Lab). Higher SCR values indicate higher sensitivity to stress [35]. For details on tasks and processing of stress markers see supplementary material.

### Statistical analyses

Descriptive and clinical characteristics were compared using t-tests and χ^2^ tests. For all analyses including controls we included education as covariate of no interest. For all analyses within patients, we additionally added duration of illness, and OLZ equivalents. Finally, all follow-up analyses were additionally corrected for symptom severity (PANSS total) at baseline. For results without covariates and justification of covariates see supplement (Supplementary Section 1.3, Supplementary Section 3). To correct for multiple comparisons, we used Sidak correction and Geiser-Greenhouse for sphericity adjustment respectively.

Our first interest was to replicate increased PS in patients with paranoia compared to patients without paranoia and healthy controls. We therefore examined group differences (patients with and without paranoia and controls) in the stop-distance and fixed-distance tasks, applying repeated measures ANCOVAs. In addition, we wanted to confirm the suggested diagnostic ability of the PS test to detect paranoia. For the stop-distance paradigm, we calculated receiver-operating curves (ROC) to determine the specificity and sensitivity of the mean PS in detecting paranoia (BPS ratings −1, −2 or −3), as well as severe paranoia (BPS ratings −2 and −3) with PS.

Next, we tested if patients with paranoia show increased levels of stress and if severity of paranoia as well as PS are associated with a marker of stress (SCRs). Therefore, we tested associations with stress for both categorical and dimensional measures of paranoia and PS. In detail, we compared differences in stress levels between healthy controls, patients with and patients without paranoia using one-way ANOVA. In addition, we investigated the relationship between severity of paranoia as well as PS and stress with partial correlations correcting for duration of illness and OLZ equivalents.

Moreover, we tested whether altered PS or stress (SCR) can predict functioning or improvement of functioning after 6 months. Thus, we performed simple linear regression analyses, partial correlations, and hierarchical regression analyses respectively. In detail, we performed linear regression with baseline PS and stress (SCR) with follow-up measures of functioning as well as with improvement of functioning. In addition, we tested if improvement of paranoia severity or PS is associated with improvement of functioning with partial correlations. Finally, to evaluate the unique contribution of PS on follow-up measures of functioning, we conducted hierarchical regression analyses. In the first step we entered covariates (duration of illness, OLZ equivalents and PANSS total) and baseline functioning measures, while in the second step we introduced the improvement in PS to determine its additional predictive value. Improvement in all measures of interest was defined as a percentage change from the baseline measures using the formula C=(x_2_–x_1_)/x_1_*100.

## Results

### Increased PS in patients with paranoia

**Fig. 1:**
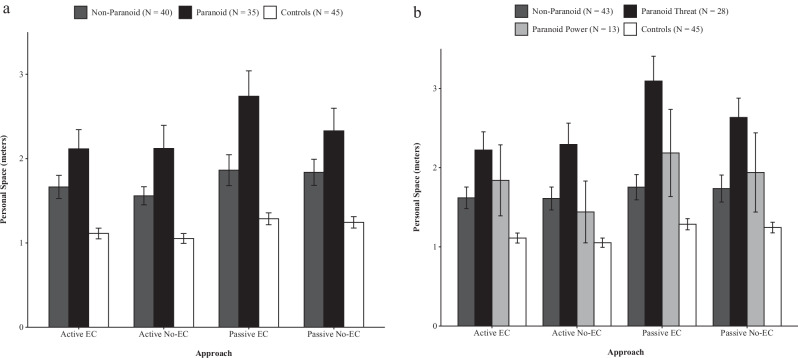
Personal space preferences in stop-distance paradigm. Mean personal space across conditions of Approach (active vs. passive) and Eye Contact (eye contact vs. no-eye contact) in stop-distance paradigm between (**a**) GPTS groups and (**b**) BPS groups. Error bars represent ± 1 SE from the mean.

Comparing PS we found a significant group effect. Patients with paranoia had higher PS compared to patients without paranoia, or healthy controls (Table 2, Fig. 1). This holds true for both Paranoia Scales. Moreover, a main effect for the condition Eye-Contact (BPS scale), an interaction between group *Approach (BPS Scale), as well as a three-way interaction group * Eye-Contact * Approach were shown (GPTS: Approach * Eye Contact * Group: F = 3.214, df = 2.0, *p* = 0.04; BPS: Approach * Eye Contact * Group: F = 2.715, df = 3.0, *p* = 0.04). Continuous associations of paranoia (GPTS-B scores, and BPS affect scores) and personal space in patients are given in the supplement (Supplementary Table 1).

**Table 2:** Personal space & VAS comfort ratings: ANCOVAs.

Personal space during the stop-distance task					
Green paranoid thought scale	*Effect*	*F*	*Df*	*P*	*Post-hoc*
Groups: Paranoid, Non-Paranoid, Controls	Group	12.985	2	**<0.001**	Paranoid > Non-Paranoid, Controls; Non-Paranoid > Controls
	Approach	0.032	1.0	0.85	
	Eye Contact	1.220	1.0	0.27	
	Approach*Group	2.061	2.0	0.13	
	Eye Contact*Group	0.657	2.0	0.52	
	Approach*Eye Contact *Group	3.214	2.0	**0.04**	

Bern psychopathology scale	*Effect*	*F*	*Df*	*P*	*Post-hoc*
Groups: Paranoid Threat, Paranoid Power, Non-Paranoid, Controls	Group	11.203	3	**<0.001**	Paranoid threat > Non-Paranoid, Controls
	Approach	0.074	1.0	0.78	
	Eye Contact	4.048	1.0	0.**04**	
	Approach*Group	3.517	3.0	0.**01**	
	Eye Contact*Group	1.912	3.0	0.13	
	Approach*Eye Contact*Group	2.715	3.0	**0.04**	

VAS comfort ratings during the fixed distance task					
Green paranoid thought scale	*Effect*	*F*	*Df*	*P*	*Post-hoc*
Groups: Paranoid, Non-Paranoid, Controls	Group	8.708	2	**<0.001**	Paranoid <Controls; Non -Paranoid < Controls
	Distance	1.294	2.0	0.27	
	Distance*Group	1.763	4.0	0.13	

Bern psychopathology scale	*Effect*	*F*	*Df*	*P*	*Post-hoc*
Groups: Paranoid Threat, Paranoid Power, Non-Paranoid, Controls	Group	5.539	3	**<0.001**	Paranoid Threat < Controls; Non -Paranoid < Controls
	Distance	1.818	1.9	0.16	
	Distance*Group	3.558	5.9	**<0.01**	

Significant *p* values are indicated in bold; covariate: education.

Moreover, ratings of comfort at fixed distances showed significant group effects (Table 2; see Supplementary Fig. 1). Specifically, patients with paranoid threat rated comfort lower than healthy controls. Additionally, the analysis revealed a significant interaction between Distance and Group (BPS). In fact, patients with paranoid threat showed significantly lower comfort at distances <1.5 meters compared to healthy controls and at 0.5 meter compared to patients with paranoid power (see Supplementary Fig. 1b).

### PS test has diagnostic ability to detect paranoia

The PS test has good discriminative value. At a personal distance of 1.1 m (cut-off of best discriminative performance of our previous study) [5], the test had a sensitivity of 92% and specificity of 57% (ROC, area under the curve = 0.81, *p* < 0.001). In addition, severe paranoia (BPS affectivity ≤ −2) was detected with 87% sensitivity and 81% specificity (*J* = 0.67 at the optimal cut-off value of 1.6 m, ROC, area under the curve = 90, *p* < 0.001) (see Supplementary Fig. 2).

### Increased PS and paranoia are associated with high stress

**Fig. 2:**
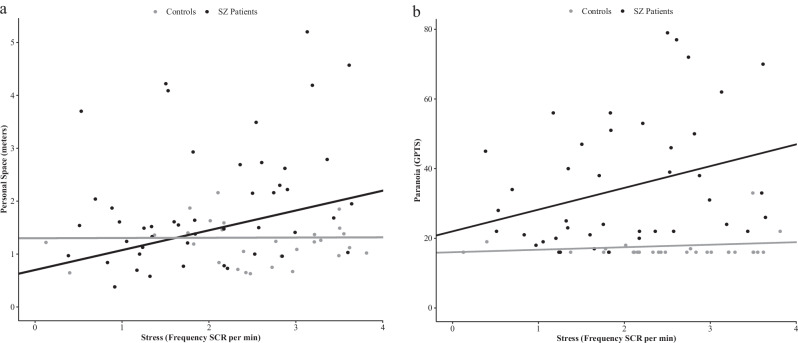
Associations of PS and paranoia with stress. Relationship (**a**) between mean personal space and stress, and (**b)** between paranoia and stress across patients and healthy controls corrected for duration of illness and OLZ within patients.

Stress parameters differed significantly across groups (GPTS). Specifically, non-paranoid patients showed reduced stress levels compared to healthy controls (Supplementary Fig. 3). Large PS (*r* = 0.32, *p* < 0.05) and severe paranoia (*r* = 0.36, *p* < 0.05) were associated with high stress in patients but not in controls (Fig. 2).

### Relationship of functioning with PS or stress

A total of 48 patients with schizophrenia (51.1%) completed the follow-up measures after 6 months. At baseline we found no differences in clinical characteristics, paranoia ratings, or PS measures between patients completing the study and patients lost to follow-up. However, patients who returned for follow-up had higher baseline levels of functioning (see Supplementary Table 2). Stress, as well as PS at baseline failed to predict follow-up measures of functioning (see Supplementary Table 3). However, stress at baseline predicted improvement in global functioning in 6 months, *R*^2^_adj_ = 0.13, *F* = 4.383, b = −0.40, *p* < 0.05. Moreover, improvement in PS was associated with improvement in social functioning (*r* = −0.35, *p* < 0.05; see Supplementary Fig. 4). This held true after controlling for the effects of covariates and baseline functioning measures (Table 3). In particular, when PS was added to the model, the model’s explanatory power improved by 9% of the total variance suggesting that changes in PS are associated with changes in the follow-up outcome measures.

**Table 3:** Hierarchical regression analyses on follow-up measures of functioning.

Dependent variable: follow-up functioning SOFAS										
	Block 1: Covariates & baseline measures					Block 2: Δ Personal space				
Independent Variables	R^2^	b	Beta	SE B	*p*	ΔR^2^	b	Beta	SE B	*p*
	0.29					0.29				
OLZ		−0.42	−0.20	0.30	0.17		−0.56	−0.27	0.29	0.06
DOI		−0.28	−0.12	0.36	0.43		−0.42	−0.18	0.34	0.22
PANSS Total		0.09	0.07	0.21	0.65		0.22	0.18	0.20	0.28
SOFAS Bas.		0.55	0.20	0.46	**0.009**		0.63	0.53	0.19	**0.002**
	0.39					0.09				
*Δ* PS							−0.10	−0.32	0.04	**0.02**
(ANOVA) Block 1:	F (4,42) = 4.051^**^					Block 2:	F (5,42) = 4.758^**^			

Significant *p* values are indicated in bold.

*OLZ* olanzapine equivalents, *DOI* duration of illness, *PANSS* positive and negative syndrome scale, *SOFAS Bas* social assessment of functioning scale at baseline, *Δ PS* Δ personal space, *Block 1* baseline variables, *Block 2* baseline variables + Δ Personal Space.

^**^p < 0.01.

## Discussion

The identification of paranoia in clinical interviews can be challenging. Here we replicated that impaired personal space (PS) regulation can identify paranoid threat with excellent sensitivity (92%) at 1.1 m, and severe paranoia with high sensitivity (87%) and specificity (81%) at 1.6 m. Patients with paranoia experience substantial discomfort at personal distances that are comfortable for healthy subjects, e.g. 1.5 m. Our results have direct implications in the clinical practice as PS may serve as a simple bedside test to detect paranoia and suggest maintaining increased PS with patients with paranoia. Furthermore, as hypothesized we demonstrate that paranoia and increased PS are associated with altered stress regulation in schizophrenia. Contrary to our hypothesis, neither PS nor paranoia at baseline predicted functional outcome after six months. However, stress markers at baseline predicted improvement of functioning, while improvement of PS was associated with improvement of social functioning. Thus, altered stress regulation may be related to reduced perceived safety and increased need of PS in paranoia. In addition, higher stress levels and persistent need of increased PS may add to unfavourable outcomes in the short-term follow-up in patients with paranoia.

The main finding of our study corroborates increased PS and reduced comfort at a fixed distance of 0.5 m in patients with paranoid threat [5]. In fact, we confirmed an increased need of PS in patients with schizophrenia, which is particularly pronounced in patients experiencing paranoid threat. Most importantly our results corroborated the PS test to have good diagnostic properties in detecting paranoia with 92% sensitivity. Thus, the PS test may be useful to screen for paranoia, replicating our own finding and complementing previous reports of increased PS in schizophrenia in general [5, 14, 16, 17, 36] and in patients with positive symptoms specifically [18, 37]. Furthermore, patients with paranoia had larger PS compared to non-paranoid patients. However, contrary to our previous report PS was also increased to some degree in non-paranoid patients. Similarly, comfort was reduced at a fixed distance of 0.5 m in non-paranoid patients compared to healthy controls. Nevertheless, our results speak towards a specific relationship of threat experience and PS expansion [14, 38, 39]. Importantly, the current data stem from a larger, independent dataset using both active and passive PS tasks. Interestingly, we noted an Approach-by-group interaction as well as as a three-way Eye-Contact-by-Approach-by-group interaction with markedly increased distances in the passive condition with eye contact in patients with paranoid threat. This interaction suggests that paranoid patients particularly suffer from PS invasion when being approached while they benefit from averted gaze during approach. Given that larger PS can be interpreted as increased safety behaviour, our results further confirm high levels of safety seeking behaviours in patients with paranoia [40, 41]. In sum, we validated increased PS in patients with paranoid threat possibly pointing to altered safety behaviour in these subjects. Furthermore, we confirm that the PS test holds diagnostic potential to detect severe paranoid threat.

Altered stress regulation may critically contribute to paranoia and PS dysregulation. Particularly social stress may increase paranoid symptoms in schizophrenia [42–44] and in youth at clinical high-risk for psychosis [45, 46]. However, measures of physiological stress markers in patients with paranoia are scarce. Thus, we tested the skin conductance responses (SCRs) per minute and its association with paranoia and PS. In patients with schizophrenia, markers of high stress were indeed associated with larger PS and more severe paranoia. Similarly, healthy controls show increase in stress markers when being stressed by physical proximity [47]. Likewise, comfort ratings during the PS experiment in our study indicated massive social stress in controls (see Supplementary Fig. 1). In general, keeping a larger PS signals avoidance or fear [39, 48]. Most importantly a mismatch of preferred and actual PS during social interaction may trigger intense stress [49–53] which might subsequently elicit or increase paranoia. Thus, our results point to a possible mechanism translating stress into altered PS regulation in schizophrenia. Moreover, findings support the assumption that impaired feeling of safety may lead to higher stress and greater use of safety behaviors in paranoia [40, 43, 54]. Notably, stress responses were higher in healthy controls than in individuals with schizophrenia irrespective of paranoia. Thus, our findings of high stress associated with larger PS and more severe paranoia must be interpreted with caution. While data on stress markers is not fully consistent, previous evidence points to blunted stress response in schizophrenia (e.g. decreased electrodermal activity and blunting of cortisol response) [55]. In fact, blunted stress response irrespective of paranoia may hamper the identification of paranoia specific effects. Future studies should focus on comprehensive batteries of physiological stress markers in paranoia.

Contrary to our hypothesis PS and paranoia at baseline did not predict outcome after six months. Thus, despite being an indicator of cross-sectional psychopathology, PS may not become a useful predictor of future functional outcomes. Nonetheless, improvement of PS was associated with improvement of social functioning. In fact, persistent increased PS may substantially hamper social interaction. Larger distance during social interaction may appear inadequate causing difficulties or even interruption of social exchange. Importantly, patients with heightened stress during the PS test faced a decline of social functioning during follow-up.

In sum, we provide fist evidence for a link between stress during PS dysregulation and poor functional outcome after six months. Thus, our results support the suggested cyclical relationship between increased need of PS, stress, and paranoia. In addition, our results support the hypothesis that social stress may be specifically predictive for social functioning in patients with paranoia [40, 56–58]. However, future studies are needed to precisely test how stress and persistent increased need of PS may contribute to social withdrawal and poor social functioning in patients with paranoia.

Some limitations require discussion. First, most of our patients were medicated, which, in principle, can alter measurements. Still, we included OLZ equivalence dosage as covariate of interest in our analyses. Second, while we were able to detect paranoia with high sensitivity at the distance of our previous study (1.1 m), specificity of the PS test at this distance was moderate in the current study. In fact, distance of patients without paranoia as well as distance of healthy controls was slightly higher compared to our previous report. One reason for the increase in personal space may be the effect of the COVID-19 pandemic on personal space as measurements of the current study were performed after the pandemic [59, 60]. Third, PS failed to detect paranoia as indicated by self-report in the GPTS. We must bear in mind that patients scoring below the paranoia cutoff on the GPTS may still present paranoid symptoms. Consequently, the GPTS cutoff is not suited to identify patients without paranoia [30]. In fact, nine subjects with severe paranoid threat according to the BPS (expert rating) scored below the GPTS paranoia cutoff (self-report). Paranoia can be difficult to assess in some individuals and may instead, by its very nature, remain concealed by patients. Fourth, our study was a naturalistic longitudinal study in which patients received treatment as usual. Thus, we cannot exclude that a particular treatment strategy may have improved social functioning. Fifth a general problem of longitudinal studies is decline in attrition rate. This may eventually lead to selection bias. However, at baseline no relevant differences emerged between patients completing the study and patients lost to follow-up. Sixth, due to relatively small sample size at follow-up findings must be interpreted with caution and require replication. Finally, personal space is influenced by several factors, including gender, age, social status, cultural norms, and psychological characteristics [4–6, 16]. Thus, future studies are needed that test the impact of these variables on paranoia.

To conclude we replicate larger personal space (PS) in patients with paranoid threat suggesting increased safety behaviours. We confirm that the PS test has diagnostic potential to detect threat. Thus, a simple bedside test of PS may be useful to screen for paranoia in schizophrenia. In addition, we demonstrated an association of stress and paranoia as well as stress and PS in patients. These results support the suggested pivotal role of stress to develop paranoia. Finally, altered stress regulation as well as persistent increased need of PS may critically contribute to social withdrawal and consequently poor social functioning in patients with paranoia

## Supplementary information

Supplementary Material

## Data Availability

Participants have not provided consent to share their health related data.
