# Supplementary material for: Aberrant personal space is associated with paranoia, altered stress regulation, and unfavourable outcomes at 6 months’ follow-up in schizophrenia
Source: Mol Psychiatry. 2025 Apr 9;30(9):4214–21. doi: 10.1038/s41380-025-02999-x (PMC12339393; doi:10.1038/s41380-025-02999-x)
Supplement: Supplementary file 1 — Supplementary Material [file 41380_2025_2999_MOESM1_ESM.docx]

**Supplementary Content**

[**Supplementary Section 1. Methods Supplement** 3](#_Toc192798180)

[*1.1.* *Procedure & Experimental Tasks* 3](#_Toc192798181)

[*1.2.* *EDA Processing* 3](#_Toc192798182)

[*1.3.* *Confounding Factors* 4](#_Toc192798183)

[**Supplementary Section 2. Results Supplement** 4](#_Toc192798184)

[*2.1 Consistency paranoia classification with GPTS and BPS* 4](#_Toc192798185)

[**Supplementary Table 1.** Means, correlation matrix for personal space measures with paranoia (GPTS-B and BPS scores) within schizophrenia patients*.* 4](#_Toc192798186)

[*2.2. Comfort ratings in Fixed-Distance groups stratified with the Green Paranoid Thought Scale (GPTS) and Bern Psychopathology Scale (BPS).* 5](#_Toc192798187)

[**Supplementary Figure 1.** Comfort ratings in Fixed-Distance paradigm between (a) GPTS groups, and (b) BPS groups. Error bars represent ± 1 SE from the mean**.** 5](#_Toc192798188)

[*2.3. Receiver Operating Curve Characteristic of Personal Space on Severe Paranoia.* 6](#_Toc192798189)

[**Supplementary Figure 2.** ROC-Curve personal space for severe paranoia among schizophrenia patients*.* 6](#_Toc192798190)

[*2.4. Mean stress measures across groups patients with and without paranoia and healthy controls.* 6](#_Toc192798191)

[**Supplementary Figure 3A.** Average stress measures between Groups of Controls and Schizophrenia patients with and without paranoia. Error bars represent ±1 SE from the mean. 6](#_Toc192798192)

[**Supplementary Figure 3B.** Average stress measures between Groups of Controls and Schizophrenia patients. Error bars represent Mean ±1 SE. 7](#_Toc192798193)

[*2.5. Association between improvement in personal space and improvement in social functioning (SOFAS).* 7](#_Toc192798194)

[**Supplementary Figure 4.** Association between improvement in personal space and improvement in social functioning (SOFAS). 7](#_Toc192798195)

[*2.6. Supplementary Outcome Findings* 8](#_Toc192798196)

[**Supplementary Table 2.** Comparison of demographic and clinical characteristics at baseline in patients who completed that study (Completers) and who were lost to follow-up (Non-completers) 8](#_Toc192798197)

[**Supplementary Table 3.** Predictors of functioning after 6 months. 8](#_Toc192798198)

[**Supplementary Figure 5.** Association between (a) follow-up measures of social functioning (SOFAS), (b) follow-up measures of global functioning (GAF) and improvement in severity of paranoia corrected for duration of illness, OLZ, and PANSS Total. 9](#_Toc192798199)

[**Supplementary Section 3. Supplementary Results without Covariates** 10](#_Toc192798200)

[3.1. *Personal space in patients with paranoia* 10](#_Toc192798201)

[**Supplementary Table 4.** Personal Space & VAS Comfort Ratings: ANCOVAs 10](#_Toc192798202)

[*3.2. Increased personal space and paranoia association with high stress.* 11](#_Toc192798203)

[*3.3. Personal space and stress measures in predicting functional outcome* 11](#_Toc192798204)

[**Supplementary Table 5.** Hierarchical Regression Analyses 11](#_Toc192798205)

## **Supplementary Section 1. Methods Supplement**

## *Procedure & Experimental Tasks*

The order of assessments began with participants’ screening to determine eligibility for the study, followed by installation of the E4 wristband, demographic assessments, personal space task, and then diagnostic and functioning assessments. Physiological recordings were measured at 3 different timepoints: (a) at baseline -15 minutes before the experimental tasks – when participants answered to demographic questions (b) during the stop-distance paradigm via presses of event buttons at the start and stop of each trial across all conditions, and (c) during the fixed distance paradigm via button presses at the beginning (2.5 meters) and end of the task (0.5 meters). All assessments were completed at baseline and after 6 months in schizophrenia patients only. There was no specific intervention within this interval. Clinical ratings were carried out by well-trained clinicians (KS, MR) blind for results of the personal space task. Interpersonal distance between the participant and the experimenter was measured after each trial with a laser distance sensor (Leica Disto D2, Leica Geosystems, Heerbrugg).

## *EDA Processing*

The wristband is equipped with multiple sensors for registering real-time electrodermal activity (EDA) by capturing changes in the electrical properties of the skin. It has an event button for registering specific events that may be associated with certain physiological signals and a 3-axis accelerometer which captures motion. To ensure reliable high-quality measurements, data acquisition was carried out at room temperature between 22⁰^C^ and 24⁰^C^. First, we used EDA Explorer ^1^ for visual inspection of the signal and for automatic artifact detection. Subsequent artifact correction was done manually with Ledalab (www.ledalab.de) based on the spline interpolation method. Then, we conducted a continuous decomposition analysis (CDA) to decompose the signal into its phasic and tonic components for both baseline and task measures ^2^. From CDA output the total number of skin conductance responses (SCRs), and the frequency of SCRs per minute were extracted. The overall clean EDA signal after preprocessing and manual artifact correction was 98.16 % for both baseline and task measures.

Before any statistical analyses EDA responses were normalized to account for any individual differences in skin conductance. Normalization was done through baseline correction and subsequent log transformations of the skewed data ^3^. For statistical analysis we considered only participants who responded to the elicited stimuli, hereafter referred to as *responders* (n_co_ = 30, n_sz_ = 52).

## *Confounding Factors*

As seen in Table 1, controls and patients did not significantly differ in age and gender but had significantly higher education years than patients. To account for confounding effects that may be associated with demographic factors we therefore included education as covariate of no interest. In analyses that included only patients we used medication as covariate to control for variations that may be related to medication effects and duration of illness to account for temporal variations due to progression of illness. Also, medication dosage significantly differed across patients with and without paranoia (*t* (73) = -3.682, *p < .*001). Moreover, symptom severity (PANSS Total) at baseline can be relevant factor influencing outcome in patients. Therefore, by including them as covariates we accounted for the potential impact of differences among patients in symptom severity.

# **Supplementary Section 2. Results Supplement**

## *2.1 Consistency paranoia classification with GPTS and BPS*

Nine subjects with severe paranoid threat according to the BPS (ratings of -2, -3, +2 or +3) scored below the GPTS-cutoff of paranoia (> 35). However, all but one subject with severe paranoia in the BPS (ratings of -2, -3, +2 or +3) had at least elevated paranoia (≥ 24 points) according to the GPTS.

### **Supplementary Table 1.** Means, correlation matrix for personal space measures with paranoia (GPTS-B and BPS scores) within schizophrenia patients*.*

|  | Correlation GPTS-B (r; p) | Correlation BPS affectivity (r; p) |
| --- | --- | --- |
| 1. Mean IPS Passive Eye-Contact (distance, meter) | **r = .31; p = .008** | **r = -.30; p = .006** |
| 1. Mean IPS Passive No Eye-Contact (distance, meter) | r = .17; p = .137 | **r = -.29; p = .008** |
| 1. Mean IPS Active Eye-Contact (distance, meter) | r = .21; p = .077 | r = -.19; p = .086 |
| 1. Mean IPS Active No Eye-Contact (distance, meter) | r = .22; p = .062 | **r = -.23; p = .035** |

IPS: Interpersonal Space; Covariates of no interest education, duration of illness, and OLZ equivalents.

## *2.2. Comfort ratings in Fixed-Distance groups stratified with the Green Paranoid Thought Scale (GPTS) and Bern Psychopathology Scale (BPS).*

| 1. **Green Paranoid Thought Scale** |
| --- |
| 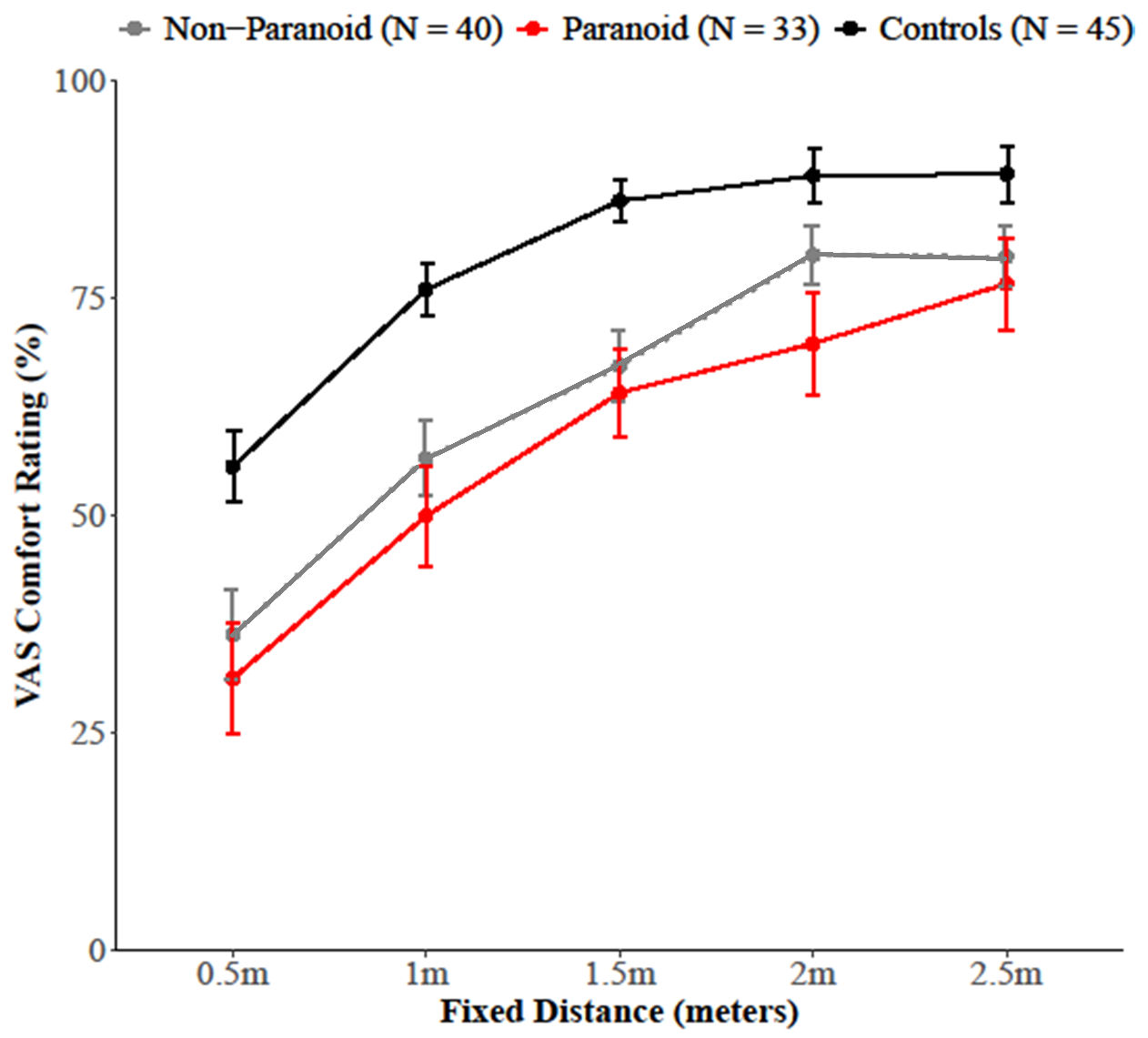 |
| 1. **Bern Psychopathology Scale** |
| 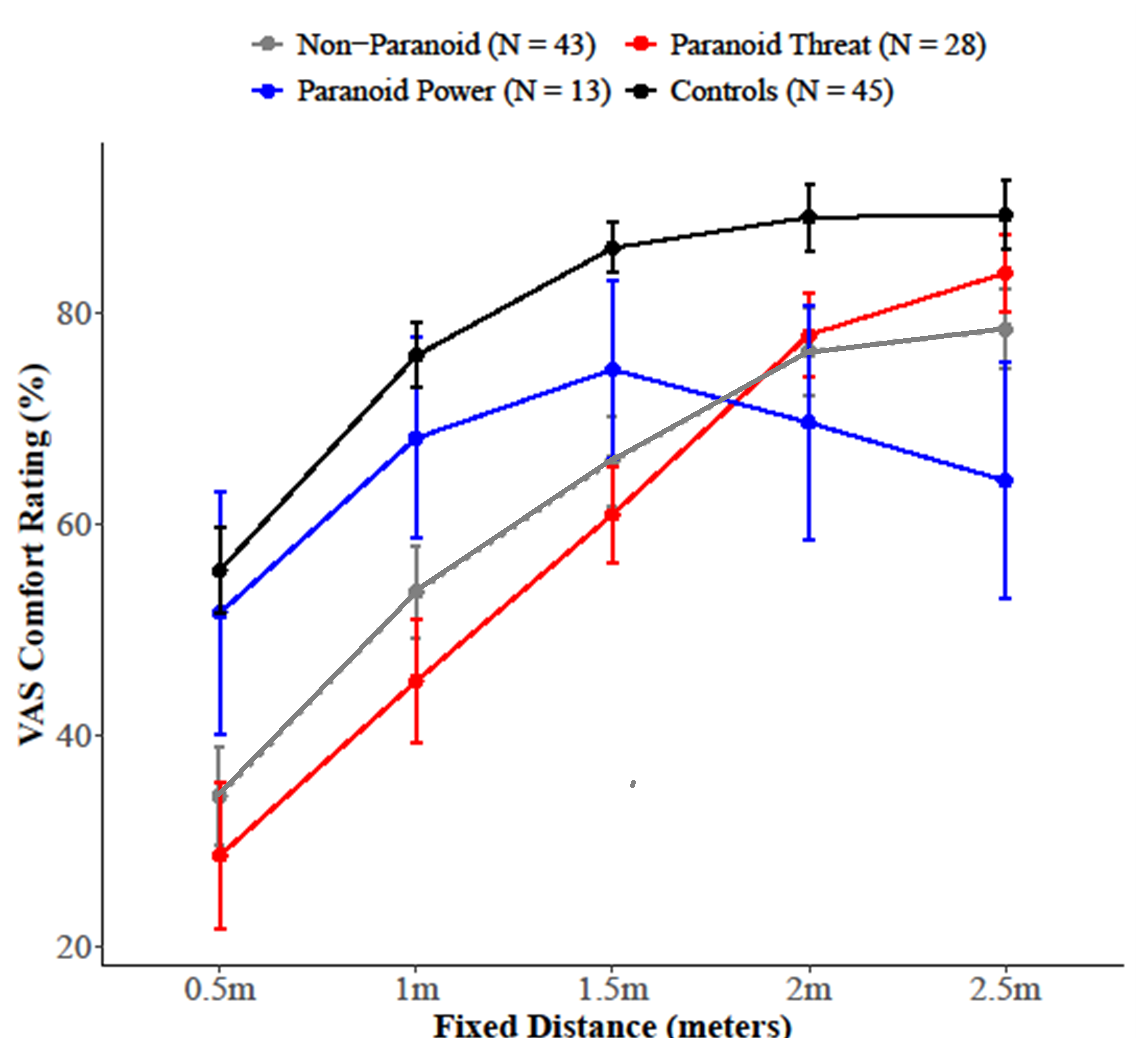 |

### **Supplementary Figure 1.** Comfort ratings in Fixed-Distance paradigm between (a) GPTS groups, and (b) BPS groups. Error bars represent ± 1 SE from the mean**.**

## *2.3. Receiver Operating Curve Characteristic of Personal Space on Severe Paranoia.*

**
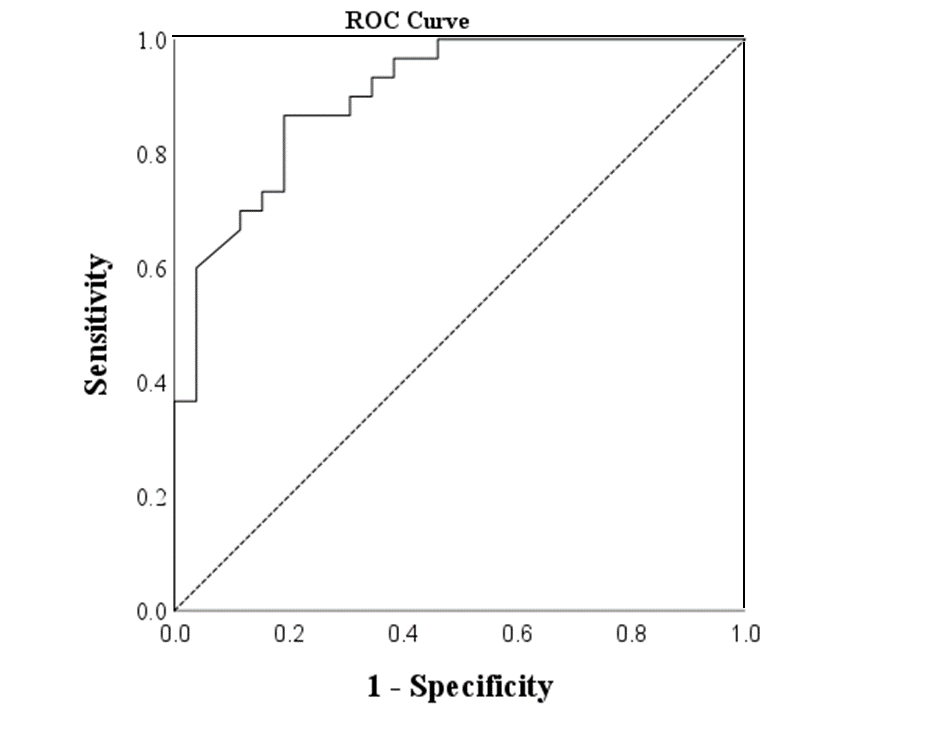
**

### **Supplementary Figure 2.** ROC-Curve personal space for severe paranoia among schizophrenia patients*.*

## *2.4. Mean stress measures across groups patients with and without paranoia and healthy controls.*

|  |  |
| --- | --- |

### **Supplementary Figure 3A.** Average stress measures between Groups of Controls and Schizophrenia patients with and without paranoia. Error bars represent ±1 SE from the mean.


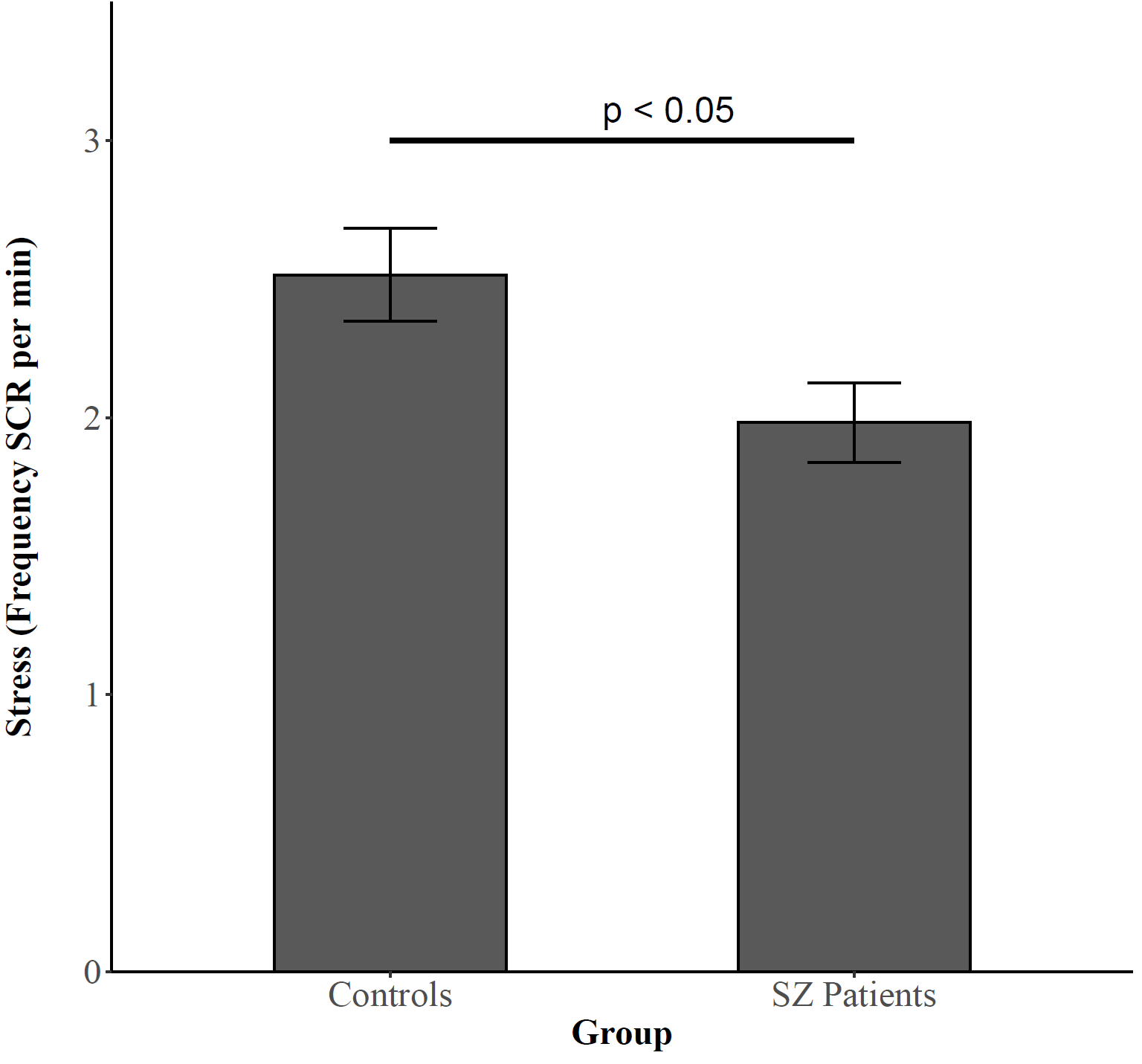


### **Supplementary Figure 3B.** Average stress measures between Groups of Controls and Schizophrenia patients. Error bars represent Mean ±1 SE.

## *2.5. Association between improvement in personal space and improvement in social functioning (SOFAS).*

**Supplementary Figure 4.** Association between improvement in personal space and improvement in social functioning (SOFAS). Improvement in personal space means a decrease in personal space. Corrected for DOI, OLZ, and PANSS Total.

## *2.6. Supplementary Outcome Findings*

### **Supplementary Table 2.** Comparison of demographic and clinical characteristics at baseline in patients who completed that study (Completers) and who were lost to follow-up (Non-completers)

| **Values** | **Completers (N= 48)** | **Non-Completers (N = 41)** | ***Χ ^2^/ t*** | ***P* value** |
| --- | --- | --- | --- | --- |
| Male, n (%) | 31 (62.50) | 23 (58.53) | .41 | .523 |
| Age, mean (SD), years | 37.21 (12.69) | 39.13 (13.01) | -.72 | .237 |
| Education, mean (SD), years | 13.44 (2.60) | 13.48 (3.12) | -.07 | .474 |
| BMI, mean (SD), kg/m^2^ | 24.67 (4.54) | 25.84 (4.71) | -1.22 | .227 |
| BPS, mean (ranks) | 43.35 (1.72) | 44.79 (.50) | .07 | .788 |
| GPTS, mean (SD) | 33.27 (17.67) | 40.00 (20.52) | -1.57 | .121  .329  **< .001**  **< .001** |
| Distance, mean (SD), m | 2.01 (1.50) | 2.34 (1.62) | -.98 |  |
| GAF, mean (SD) | 47.07 (14.91) | 36.82 (13.58) | 3.41 |  |
| SOFAS, mean (SD) | 47.49 (16.19) | 35.78 (12.26) | 3.90 |  |
| OLZ, mean (SD), mg | 15.38 (11.47) | 12.69 (10.44) | 1.19 | .236 |
| Illness Duration, mean (SD), years | 8.96 (8.60) | 9.86 (9.53) | -.47 | .640 |

*Note:* BMI: body mass index; GPTS: Green Paranoid Thoughts Scale; GAF: Global Assessment of Functioning scale; SOFAS: Social Assessment of Functioning Scale; OLZ: olanzapine equivalents. Significant p values are indicated in bold.

### **Supplementary Table 3.** Predictors of functioning after 6 months.

| *Baseline* | *Follow-up* | *R*^2^_adj_ | *F* | B | *p* |
| --- | --- | --- | --- | --- | --- |
| Stress | SOFAS | .02 | 1.516 | -.26 | .23 |
| Stress | GAF | .01 | 1.405 | -.25 | .25 |
| PS | SOFAS | -.02 | .277 | -.08 | .60 |
| PS | GAF | -.02 | .06 | -.04 | .80 |
| *Baseline* | *Improvement (Δ)* |  |  |  |  |
| Stress | SOFAS | .00 | 1.060 | -.22 | .31 |
| Stress | GAF | .13 | 4.383 | .40 | **.04** |
| PS | SOFAS | -.01 | .340 | -.09 | .56 |
| PS | GAF | -.01 | .319 | -.08 | .57 |

*Note:* GAF = Global Assessment of Functioning scale , SOFAS = Social Assessment of Functioning Scale, PS = Personal Space. Significant p values are indicated in bold.

Improvement in paranoia severity was associated with follow-up measures of social (*r* = -.42, *p* <.05;Figure S5a) and global functioning (*r* = -.51, *p* <.01;Figure S5b).

| **a.** | **b**. |
| --- | --- |
|  |  |

### **Supplementary Figure 5.** Association between (a) follow-up measures of social functioning (SOFAS), (b) follow-up measures of global functioning (GAF) and improvement in severity of paranoia corrected for duration of illness, OLZ, and PANSS Total.

# **Supplementary Section 3. Supplementary Results without Covariates**

## 3.1. *Personal space in patients with paranoia*

##

### **Supplementary Table 4.** Personal Space & VAS Comfort Ratings: ANCOVAs

| **Personal Space during the Stop-Distance Task** | | | | | | | | | |
| --- | --- | --- | --- | --- | --- | --- | --- | --- | --- |
|  | *Effect* | | *F* | *Df* | | *P* | | *Post-hoc* | |
| **Green Paranoid Thought Scale** | | | | | | | | | |
| Groups: Paranoid, Non-paranoid, Controls | Group | | 15.035 | 2 | | **<.001** | | Paranoid > Non –Paranoid, Controls;  Non –Paranoid > Controls | |
|  | Approach | | 23.465 | 1.0 | | **<.001** | |  | |
|  | Eye Contact | | 4.463 | 1.0 | | **.03** | |  | |
|  | Approach*Group | | 1.431 | 2.0 | | .24 | |  | |
|  | Eye Contact*Group | | .880 | 2.0 | | .42 | |  | |
|  | Approach*Eye Contact*Group | | 3.105 | 2.0 | | **.04** | |  | |
| **Bern Psychopathology Scale** |  | |  | |  | |  | |  |
| Groups: Paranoid Threat, Paranoid Power, Non-paranoid, Controls | Group | | 12.900 | | 3 | | **< .001** | | Paranoid threat > Non-Paranoid, Controls |
|  | Approach | | 22.489 | | 1.0 | | **< .001** | |  |
|  | Eye Contact | | 8.703 | | 1.0 | | **.004** | |  |
|  | Approach*Group | | 3.345 | | 3.0 | | **.02** | |  |
|  | Eye Contact-Group | | 1.901 | | 3.0 | | .13 | |  |
|  | Approach*Eye Contact*Group | | 2.514 | | 3.0 | | .06 | |  |
| **VAS Comfort Ratings During the Fixed Distance Task** | | | | | | | |  | |
|  | *Effect* | *F* | | *Df* | | *P* | | *Post-hoc* | |
| **Green Paranoid Thought Scale** | | | | | | | | | |
| Groups: Paranoid, Non-paranoid, Controls | Group | 11.383 | | 2 | | **< .001** | | Paranoid < Controls;  Non -Paranoid < Controls | |
|  | Distance | 92.560 | | 1.9 | | **< .001** | |  | |
|  | Distance*Group | 1.415 | | 3.9 | | .23 | |  | |
| **Bern Psychopathology Scale** |  |  | |  | |  | |  | |
| Groups: Paranoid Threat, Paranoid Power, Non-paranoid, Controls | Group | 7.872 | | 3 | | **< .001** | | Paranoid Threat <  Controls;  Non-Paranoid, Controls | |
|  | Distance | 67.303 | | 1.9 | | **< .001** | |  | |
|  | Distance*Group | 3.551 | | 5.9 | | **.002** | |  | |

*Note:* significant p values are indicated in bold.

## *3.2. Increased personal space and paranoia association with high stress.*

When we removed the covariates, the analysis showed that paranoia and interpersonal space were positively associated with stress in patients, *r* = .33, *p* < .05 and *r* = .32, *p* < .05 respectively.

## *3.3. Personal space and stress measures in predicting functional outcome*

The relationship between stress and improvement in global functioning remained significant after removing the covariates *r* = -.49, *p* <.05. In contrast, the significant relationship between improvement in PS and improvement in social functioning was no longer present, *r* = -.21, *p* =.16. Likewise, the relationship between improvement in paranoia severity and follow-up measures of social and global functioning was no longer significant SOFAS: *r* = -.24, *p* = .14 and GAF: *r* = -.26, *p* =.10 respectively. Notably, improvement in personal space remained significant in predicting follow-up measures of functioning after removing the covariates from the hierarchical regression analysis (Table S4). However, removing the covariates from the model led to a decrease in the explained variance (4%) compared to the model that included covariates (9%).

### **Supplementary Table 5.** Hierarchical Regression Analyses

|  | *Block 1* | | | | | | | |  |  | *Block 2* | | | | | |
| --- | --- | --- | --- | --- | --- | --- | --- | --- | --- | --- | --- | --- | --- | --- | --- | --- |
|  | | *R*^2^ | *b* | *Beta* | *SE B* | *P* | |  |  | *ΔR*^2^ | *b* | *Beta* | *SE B* | *p* |  |  |
| MODEL 1 | | .23 |  |  |  |  | |  |  | .23 |  |  |  |  |  |  |
| SOFAS Bas. | |  | .57 | .48 | .16 | **<.001** | |  |  |  | .61 | .51 | .15 | **< .001** |  |  |
| MODEL 2 | | .27 |  |  |  |  | |  |  | .04 |  |  |  |  |  |  |
| *Δ* PS | |  |  |  |  |  | |  |  |  | -.05 | -.19 | .04 | **< .001** |  |  |
| Model (ANOVA) | |  | *F* (1,43) = 13.004*** | | | |  |  |  |  | *F* (2,43) = 7.738** | | | |  |  |

*Note:* SOFAS Bas. = Social Assessment of Functioning Scale at Baseline, Δ PS = Δ Personal Space. Significant p values are indicated in bold; ** p < .05, ** p < .01, *** p < .001.*

**References**

1. Automatic identification of artifacts in electrodermal activity data. *Proceedings of the 2015 37th Annual International Conference of the IEEE Engineering in Medicine and Biology Society (EMBC)*2015. IEEE.

2. Benedek M, Kaernbach C. A continuous measure of phasic electrodermal activity. *Journal of neuroscience methods* 2010; **190**(1)**:** 80-91.

3. Venables PH, Christie MJ. Electrodermal activity. *Techniques in psychophysiology* 1980; **54**(3).
